# Impact of catheter angle on microsphere distribution in transarterial radioembolization: an in silico study based on ex vivo porcine liver data

**DOI:** 10.1007/s10237-026-02115-0

**Published:** 2026-09-21

**Authors:** Tess J. Snoeijink, Tristan G. Vlogman, Anne van den Brekel, Jan L. van der Hoek, Joey Roosen, Erik Groot Jebbink, Kartik Jain, J. Frank W. Nijsen

**Affiliations:** 1 Department of Medical Imaging, Radboud University Medical Center, Nijmegen, The Netherlands; 2 Multi-Modality Medical Imaging Group, University of Twente, TechMed Center, Enschede, The Netherlands; 3 Faculty of Engineering Technology, Department of Thermal and Fluid Engineering, University of Twente, Enschede, The Netherlands

**Keywords:** Radioembolization, Microspheres, Distribution, Ex vivo, Computational fluid dynamics

## Abstract

Computational fluid dynamics (CFD) simulations were conducted to explore the impact of catheter angle on microsphere distribution in transarterial radioembolization (TARE). The models were designed to closely replicate conditions from previously conducted ex vivo machine-perfused porcine liver experiments and therefore improve the perspective on microsphere distribution patterns. TARE with holmium-loaded microspheres was performed on an ex vivo machine-perfused porcine liver. Catheter position was imaged using angiography and ultrasound, the hepatic arterial vasculature using 3D cone beam CT, and lobe-specific microsphere distributions using holmium-sensitive T2*-weighted MRI scans. An in-house CFD model based on lattice Boltzmann and discrete element methods was developed. Simulations were parameterized with ex vivo porcine liver geometry and flow conditions as input. To further explore the role of catheter angles beyond experimental findings, four catheter angles (0°, 15°, 30°, and 45°), gradually directed toward the hepatic arterial wall, were evaluated using CFD. Ex vivo ultrasound imaging showed that the catheter was oriented at an angle of 25.2° toward the hepatic arterial wall. The simulations revealed that catheter angle strongly influences microsphere distribution, particularly in the branches closest to the injection position (lobe 3–5). The correlation between ex vivo and simulated microsphere distributions increased with catheter angle: from poor at 0° (r = 0.123), to moderate at 15° (r = 0.511), to very strong at 30° and 45° (r = 0.809 and r = 0.807, respectively). A catheter angle directed toward the hepatic arterial wall can affect microsphere distribution. In current clinical practice, the radial catheter position is not routinely visualized, complicating the reproducibility of catheter tip positioning between scout and therapeutic doses. Future research could focus on improved catheter tip visualization and the development of more stable catheter designs to enhance the reproducibility and efficacy of TARE.

**Supplementary Information:**

The online version contains supplementary material available at https://doi.org/10.1007/s10237-026-02115-0.

## Introduction

Transarterial radioembolization (TARE) has emerged as a safe and effective treatment option for patients with both primary and secondary liver cancer (Reig et al. 2022; Weber et al. 2022). During TARE, radioactive microspheres are injected into the hepatic arterial (HA) vasculature. The microspheres are transported via the blood toward the tumoral bed, where they lodge and emit their radiation (Reinders et al. 2019). TARE is most commonly performed using yttrium-90 microspheres; however, holmium-166 microspheres have also been extensively evaluated in clinical studies. Prospective trials and subsequent clinical studies have demonstrated the safety, feasibility, and efficacy of holmium-166 radioembolization in patients with primary and metastatic liver malignancies, while also enabling quantitative imaging-based treatment planning and dosimetry through SPECT/CT and MRI (Prince et al. 2018; Reinders et al. 2022; Stella et al. 2022). Although TARE is widely used, there is still a need for further optimization of tumor to non-tumor (T/N) ratios, as it has been established that this can improve survival and disease control (Garin et al. 2021; Hermann et al. 2020).

One to two weeks before TARE, a scout dose is administered to determine optimal catheter positions for the therapeutic procedure and to calculate the amount of liver–lung shunting (Ramdhani et al. 2024). This scout dose may consist of either technetium-99 m-macroaggregated albumin (^99m^Tc-MAA) or a non-therapeutic dose of holmium-166 microspheres. Variations in injection parameters such as injection rate and profile are known to affect microsphere distribution (Basciano et al. 2010; Caine et al. 2017), and it has been questioned whether this limits the predictive accuracy of the scout dose (Snoeijink et al. 2025b). In addition, variations in catheter position and angle can affect microsphere distribution (Aramburu et al. 2016b, 2017b; Basciano et al. 2010; Bomberna et al. 2021; Childress et al. 2012). In clinical practice, catheter placement relies on 2D planar imaging, which does not provide information regarding the catheter’s radial (cross-sectional) orientation. As a result, accurate replication of catheter positioning between the scout and therapeutic administration is challenging. This may contribute to the sometimes observed limited predictive accuracy of the scout dose on the final microsphere distribution (Wondergem et al. 2013).

In silico studies offer the potential to provide more insight into the impact of the catheter position on the microsphere distribution (Aramburu et al. 2022). Computational fluid dynamics (CFD) models can simulate the hepatic hemodynamics and predict flow patterns and microsphere trajectories. To compare CFD simulation results with actual in vivo microsphere distributions*,* several properties need to be extracted, such as the patient-specific geometry, blood flow rates to different areas of the liver, catheter tip location, and microsphere injection rate; properties that remain hard to retrieve in clinical practice. In the context of TARE, in vivo validation of CFD simulations remains limited. A retrospective study by Antón et al. (Antón et al. 2021) is the first one demonstrating that a CFD model successfully can predict segment-to-segment microsphere distribution. However, they also report on the misinterpretation of the exact catheter tip position.

Compared to in vivo approaches, an ex vivo model offers more controlled conditions. It enables extraction of detailed data often difficult to obtain in a clinical setting, while still closely replicating in vivo structure and function (Gravante et al. 2011). Our ex vivo machine perfusion model (Snoeijink et al. 2025a) allows detailed assessment of axial and radial catheter positions through angiography and ultrasound imaging. Additionally, as the perfusion model is MRI compatible, it also enables high-resolution holmium-based dosimetry, providing more accurate segmental microsphere distribution analysis compared to the clinically used post-radioembolization SPECT/CT (Smits et al. 2013). Despite the advantages of ex vivo models, their exact replication in a computational model still remains challenging due to the involvement of a number of biological factors that cannot be accurately modeled.

This study investigates how catheter angle affects microsphere distribution during TARE via CFD simulations. The model is parameterized using ex vivo porcine liver data (machine-perfused) and incorporates anatomical and catheter-position details, within the accuracy limits of imaging and modeling. First, we demonstrate qualitative correspondence between ex vivo and in silico results. Then, by systematically varying the catheter tip angle relative to the hepatic artery wall, we assess whether this single parameter can bring simulation outputs closer to experimental observations.

Although quantitative validation is pending and would warrant improved experimental protocols and model complexity, our results suggest catheter angle is a key parameter for aligning in silico and ex vivo experiments. Variations in microsphere distributions across different catheter angles further suggest that clinical control of this parameter could improve dosage delivery efficiency to liver tumors.

## Materials and methods

### Part (1) TARE procedure of an ex vivo perfused porcine liver under MRI

#### Ex vivo perfusion

A porcine liver, retrieved from a local slaughterhouse, was subjected to normothermic machine perfusion. The liver procurement protocol and details on the machine perfusion setup have already been described (Snoeijink et al. 2025a). The HA was cannulated indirectly through the aorta, while the portal vein (PV) was cannulated directly, both using a 25 Fr cannula. After ligation of all side branches and weighing of the liver (Hendi weighing scale, De Klomp, The Netherlands), the liver was connected to the perfusion setup. The HA blood flow rate was gradually increased to 0.22 ml/min/g liver tissue and the PV flow rate to 0.66 ml/min/g liver tissue at normothermic porcine level, i.e., 38 °C (Maione et al. 2018).

Perfusion through the HA side was performed using the Xenios NovaLung iLA ACTIVVE console (Medos Medizintechnik, Stolberg, Germany) with a DP3 pump head, generating pulsatile flow as sine waves with an amplitude equal to 20% of the mean flow rate and a frequency of 60 beats per minute. Perfusion through the PV side was performed using a Liver Assist device (XVIVO, Gothenburg, Sweden) with flow-driven settings, generating a continuous flow.

#### Imaging

First, the perfusion setup was placed in the hybrid operating room, equipped with an ARTIS pheno robotic C-arm (Siemens Healthineers, Erlangen, Germany). A 2.7 Fr/0.90 mm microcatheter (Progreat, Terumo Europe, Leuven, Belgium) was advanced into the HA vasculature under angiography guidance via an MBA™ hemostasis valve (Merit Medical Systems Inc., South Jordan, USA). The catheter was placed approximately two centimeters before the point of division into five branches supplying each liver lobe. After that, an iodine-based contrast agent (Iomeron® 300 mg/ml, Bracco Imaging, Milan, Italy) was administered (60 ml, 3 ml/s) through a three-way valve connector attached to the aortic cannula. Simultaneously, a cone beam CT (CBCT) scan with a spatial resolution of 0.64 mm was acquired to capture the 3D geometry of the HA liver vasculature.

In the subsequent step, the perfusion setup was transferred to a nearby 1.5 T MAGNETOM Aera MRI (Siemens Healthineers, Erlangen, Germany). The liver was placed within a portable, custom-modified, MRI-compatible organ chamber filled with porcine blood. This chamber was initially positioned outside the 5-gauss safety zone within the MRI room, while already connected to the pumping units located in the MRI control room via eight meters of tubing. Ultrasound imaging was then performed using an ACUSON S3000 Ultrasound System (Siemens Healthineers, Erlangen, Germany), equipped with a 14L5 linear transducer (frequency: 14 MHz, imaging depth: 1 cm). The transducer was positioned directly on the HA, and both top- and side-view images were acquired to verify the axial catheter position and to determine the radial position of the catheter in two directions. Following ultrasound acquisition, the liver was placed inside the MRI scanner for further imaging.

The MRI acquisition protocol started with a T1 VIBE DIXON anatomical scan. To analyze liver perfusion, dynamic MRI scanning of the hepatic volume was performed over time (200 series, interval of 0.86 s), during and after the arterial injection of a gadolinium-based contrast agent (Dotarem® 0.5 mmol/ml, Guerbet, Roissy CDG, France), with an injection rate of 0.3 ml/s (6 ml). After that, holmium-sensitive T2*-weighted scans were acquired before and after administering microspheres.

#### Microsphere administration

Non-radioactive holmium-165 loaded microspheres (density 1.4 g/mL (Reinders et al. 2019), mean size 30 µm (± 5 µm) (Terumo Europe NV, Safety and performance information—QuiremSpheres™. LC-80043 [09], Issue date: 2024.) were administered in five fractions of 250 mg to the liver via the conventional administration device including a V-vial (Quirem Medical B.V., Deventer, The Netherlands). For the microsphere administration, two syringe pumps (NE-4000 Multi-Phaser, New Era Pump Systems Inc., Farmingdale, USA) were used, to establish reproducible injections. Injections were performed with pulses of 0.1 ml and 32 ml/min, followed by a 0.3 s pause after each pulse. For microsphere agitation in the V-vial, 25 pulses of 0.1 ml were applied (2.5 ml), and for flushing of the administration device, 100 pulses of 0.1 ml were applied (10 ml), following the instruction for use for QuiremSpheres™ (Terumo Europe NV, Safety and performance information—Manual—QuiremSpheres™. LC-80072 [05], Issue date: 2023.).

#### Image analysis

The perfusion rates per liver lobe were determined from the dynamic contrast-enhanced MRI scans. Liver lobes were defined as: left lateral lobe, left medial lobe, right medial lobe, right lateral lobe, and caudate lobe, later referred to as lobes 1 to 5, respectively (Court et al. 2003). The T1 VIBE DIXON anatomical scan was resampled toward the dimensions of the dynamic scan via custom MATLAB acquisition code (MATLAB R2022a, MathWorks, Natick, Massachusetts, USA). In this resampled anatomical scan, regions of interest (ROIs) were delineated for the five liver lobes. For each ROI, time-intensity curves (TICs) were calculated with the percent enhancement formula PE = 100*(S_1, lobe_- S_0, lobe_)/S_1, lobe_ (Jones et al. 2013). S_0, lobe_ is the mean signal intensity in a lobe before contrast injection, and S_1, lobe_ is the signal intensity at all post-injection image acquisition points. As a measure for the perfusion rate, the maximum slope of the wash-in period of the TICs was used (Václavík et al. 2022). The perfusion rate for each liver lobe was expressed as a percentage of the total HA perfusion (0.22 ml/min/g liver tissue).

The holmium-sensitive T2*-scans were used to perform MRI-voxel-based dosimetry. A fictive specific activity of 12 MBq/mg was applied. The scans were processed in Q-suite (version 2.2, Quirem Medical B.V., Deventer, The Netherlands), and the fictive administered activity (MBq) was calculated for each liver lobe and expressed as a percentage of the total administered activity.

A reconstruction of the HA liver vasculature was retrieved by using threshold-based segmentation (353 Hounsfield Units) in 3D Slicer (version 5.0.3 (Fedorov et al. 2012)). The segmentation was further refined in Materialise Mimics (version 26.0, Leuven, Belgium), using built-in post-processing tools, including surface smoothing and hole-filling algorithms.

### Part (2) CFD simulations of the ex vivo perfused porcine liver

The CFD simulations were performed using an in-house developed coupled lattice Boltzmann (LBM) and discrete element method code. This code was described in previous work (Vlogman and Jain 2024). The solver is developed within the adaptable poly engineering simulator (APES) framework (Roller et al. 2011), which consists of a toolchain of solvers ranging from mesh generator Seeder to the LBM solver *Musubi* (Hasert et al. 2014). The solvers have been previously tested for large-scale supercomputing on major European and Japanese systems (Masilamani et al. 2014) and have been open-sourced under: https://github.com/apes-suite. The *Seeder* reads the surface of the geometry in STL format and creates a volume mesh which is written to disk. Computations are then performed by the *Musubi* LBM solver.

The reconstructed HA liver vasculature was used as a basis for the geometry in the simulations. To reduce computational cost, selected distal outlet vessels were truncated, ensuring that truncation occurred distal to the point of division into branches supplying distinct liver lobes. In case the truncation had to occur in a tortuous part of the geometry, the outlets were extended by a straight tube to promote fully developed flow at the outlets and reduce the effect of truncation on the simulation results.

The geometry was discretized using a mesh size of 5.2 × 10^–2^ mm, which is 1/12 of the catheter inner diameter (0.65 mm) and was based on the mesh refinement study in an idealized liver geometry (Vlogman et al. 2025). This led to a total of approximately 43 million elements in the mesh. A time step of 6.0 × 10^–6^ s was used to maintain accuracy and stability. The fluid inside the domain was considered incompressible with a viscosity of 3.5 × 10^–3^ Pa s and a density of 1111.8 kg/m^3^ to mimic the properties of blood (Brindise et al. 2018). Due to the high shear rates involved, blood was approximated as Newtonian fluid (Errill 1969) with a constant dynamic viscosity. While blood exhibits non-Newtonian, shear-thinning characteristics at low shear rates, it reaches a well-documented Newtonian plateau at shear rates exceeding 100 s^−1^ (Cho & Kensey 1991; Cho and Kensey 1991). In the hepatic arterial tree and the main branches under investigation, the characteristic vessel diameters (typically ranging from 1.5 to 4.0 mm) combined with the physiological flow rates driven by our boundary conditions yield characteristic shear rates well above this threshold throughout the majority of the cardiac cycle. Furthermore, for high-velocity, momentum-driven applications such as catheter-based microsphere delivery, the bulk transport dynamics and localized flow splitting are predominantly governed by geometry-induced pressure drops and inertial forces, rendering the impact of non-Newtonian viscosity variations in the main lumen negligible (Buchanan et al. 2000; Cho & Kensey 1991)(Buchanan et al. 2000; Cho and Kensey 1991).

#### Flow split

A pulsatile inlet waveform, resembling the ex vivo conditions (pulsatile flow as sine waves, frequency of 60 beats per minute), was applied to the CFD model. To approximate the ex vivo flow distribution, pressure boundary conditions were imposed at the outlets (Junk and Yang 2011). These pressures were estimated using a 0-D Poiseuille flow model to calculate the pressure drop from the inlet to each outlet, based on average vessel diameters extracted from 3D Slicer. No-slip conditions (indicating zero-velocity) were prescribed at the vessel walls using the bounce-back boundary condition in the LBM (Ladd 1994), causing incoming particles to rebound from the vessel wall.

#### Catheter configurations

CFD simulations were performed at the axial catheter position corresponding to that observed ex vivo. Curved catheter geometries were constructed such that the catheter tip is oriented at a specified angle (0°, 15°, 30°, or 45°) with respect to the vessel wall whereas far upstream from the tip the catheter is parallel to the vessel. To systematically generate smooth curves satisfying these criteria, cubic Beziér curves were used to define the centerline geometry of the catheters.

#### Microsphere injection

To simulate solid particles, a two-way coupled model was used in which the fluid affects the particle trajectories and the particles affect the fluid motion. Since the suspension of microspheres is dilute (the largest solid volume fraction is 2.7%), the effect of the volume of particles on the fluid was neglected.

**Fig. 1:**
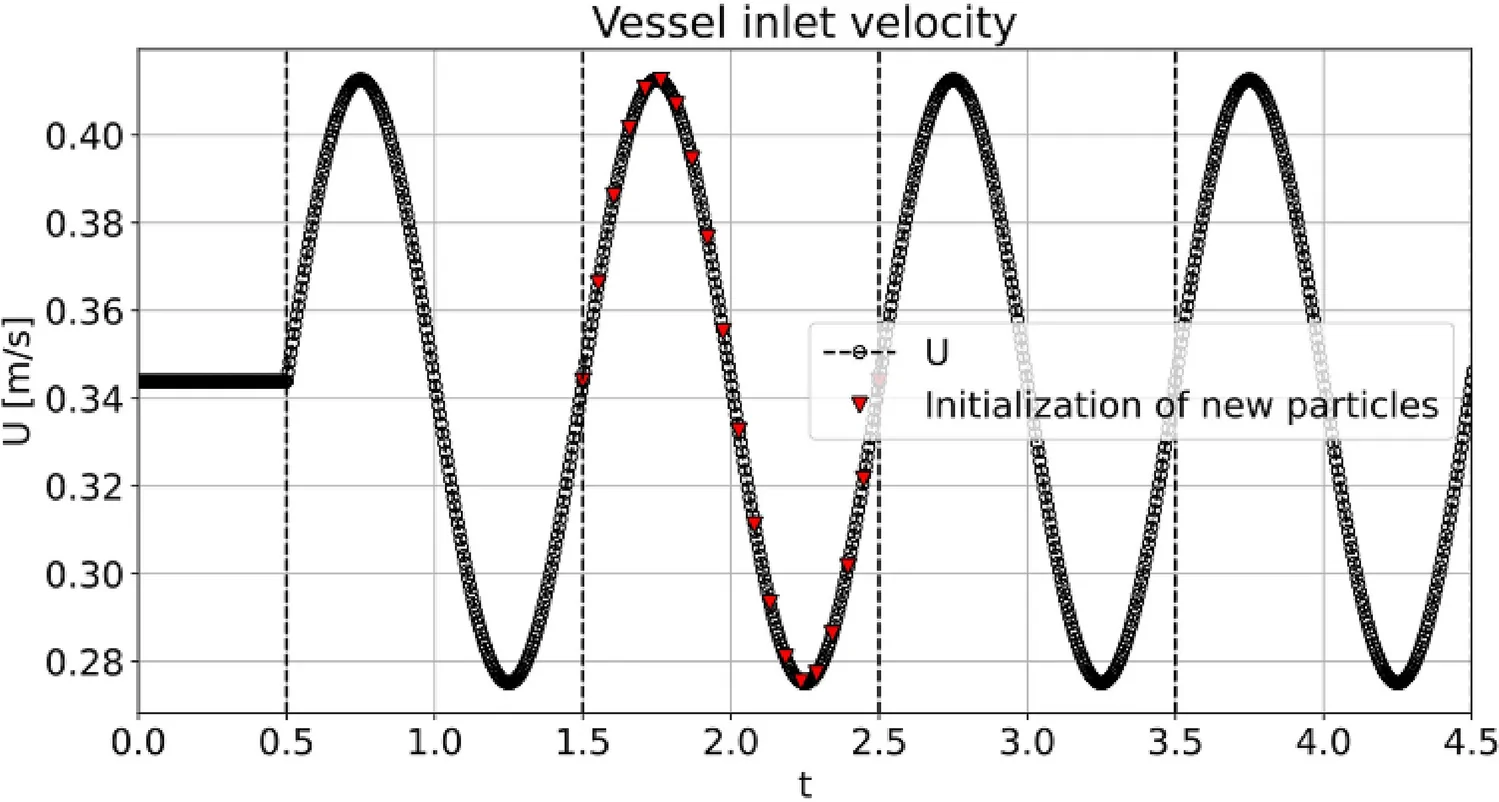
The time-varying average inlet velocity used for the CFD simulations

Each simulation starts by simulating 0.5 s of steady flow using the average HA blood flow rate, followed by one period (consisting of T = 1.0 s) of the sinusoidally varying input waveform described in the ex vivo experiment. This initial simulation is used to obtain a physically realistic initial condition for the subsequent particle simulation. During the second period, particles are injected through the catheter in a pulsed manner with pulses of 0.042 ml and 10 ml/min followed by a 0.25 s pause after each pulse. Subsequently, two additional periods were simulated with pulsatile flow maintained through the catheter to allow previously injected particles to reach the outlets. No new particles were introduced during this phase to reduce computational cost. The flow waveform used for the CFD simulations and the moments of particle injection are shown in Fig. 1. Using 1024 CPU cores of the Dutch national supercomputer *Snellius,* each particle simulation took approximately 12 h.

#### Statistical analysis

To determine correlations between the ex vivo microsphere distribution and CFD simulations, the Pearson’s correlation coefficient (r) was used, with 0 ≤ r < 0.1 none, 0.1 ≤ r < 0.3 poor, 0.3 ≤ r < 0.6 fair, 0.6 ≤ r < 0.8 moderate, 0.8 ≤ r < 1 very strong, r = 1 perfect correlation (Akoglu 2018). The data were processed using SPSS statistical software package version 29 (SPSS Inc. Chicago, IL, USA).

## Results

### Part (1) TARE procedure of an ex vivo perfused porcine liver under MRI

#### Ex vivo* perfusion*

The liver weight before perfusion was 2638 g. To achieve a flow rate of 0.22 ml/min/g liver tissue, the corresponding mean HA pump setting was 580 ml/min and mean PV pump setting was 1740 ml/min.

#### Imaging

**Fig. 2:**
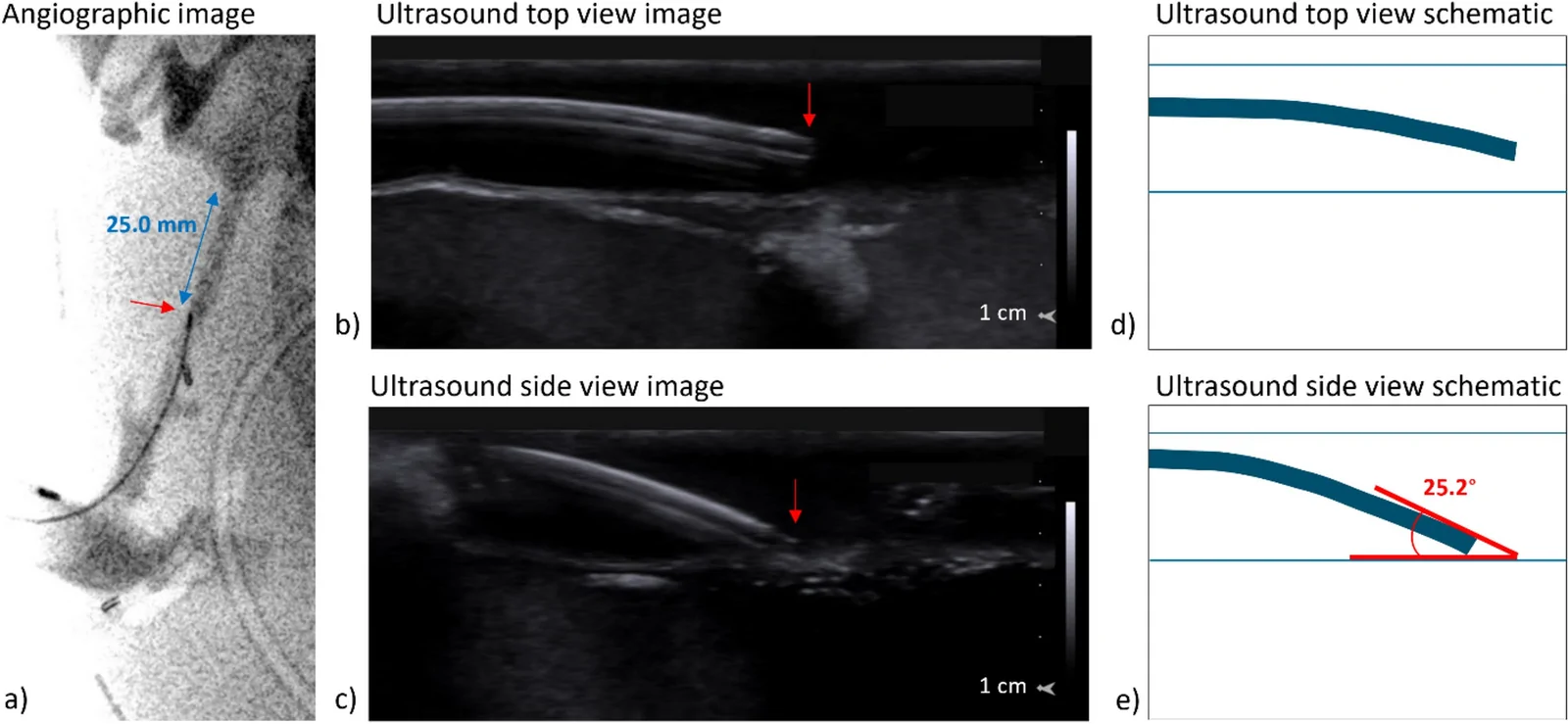
Catheter position in the hepatic artery prior to transarterial radioembolization. **a** Angiographic image, showing the axial placement (25.0 mm from the point of division into five branches). **b**, **c** Ultrasound top- and side-view images of the radial catheter location. **d**, **e** Schematics of the ultrasound images, with **e** showing the maximum catheter angle (25.2°). The red arrows indicate the catheter tip

The axial and radial catheter position during the TARE procedure was determined using both angiographic and ultrasound imaging, as illustrated in Fig. 2. The axial position of the catheter was measured at 25.0 mm proximal to the point of division into five HA branches. Ultrasound imaging further revealed that the catheter tip was oriented toward the vessel wall, with a 25.2° angle observed in the side view.

#### Image analysis

**Fig. 3:**
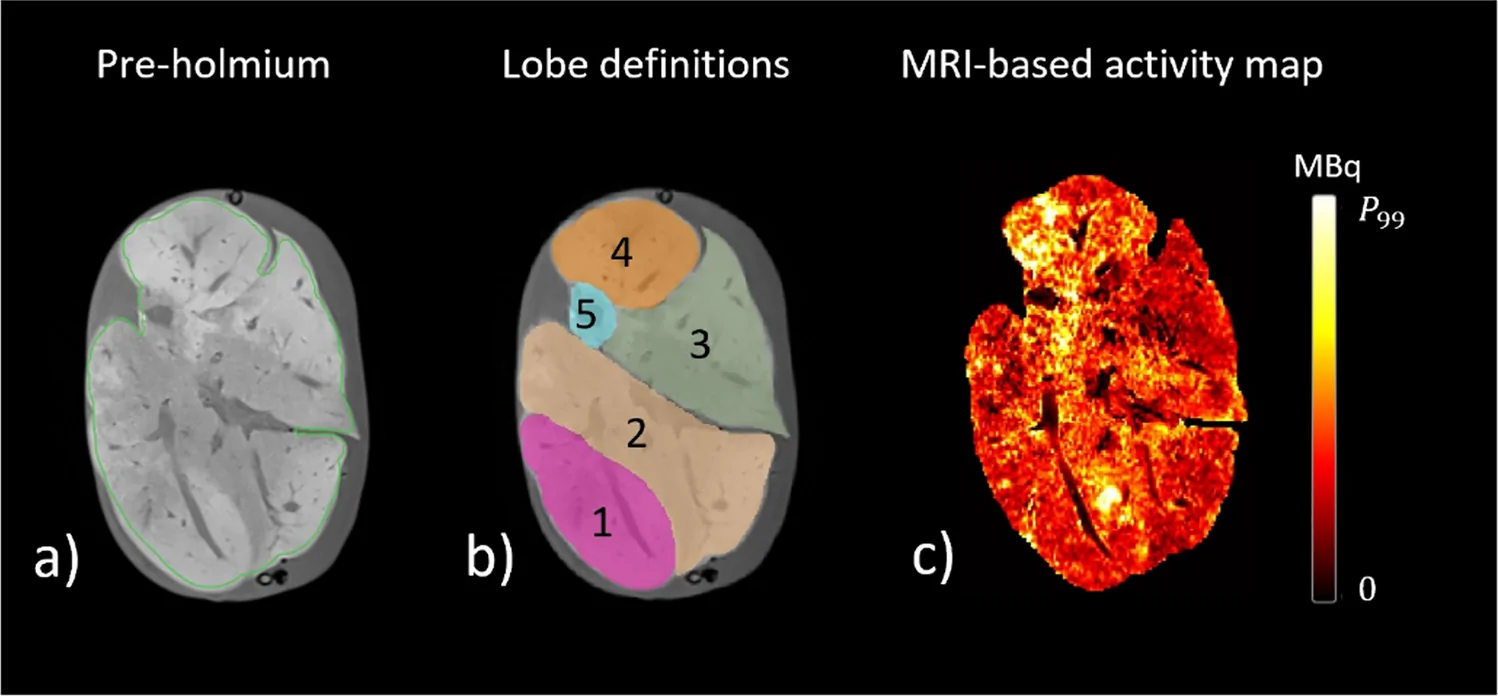
**a** Anatomical MRI scan of the ex vivo liver, **b** Lobe definitions, and **c** MRI-based activity map after administering 1250 mg of microspheres. The activity map was scaled to a maximum corresponding to the 99th percentile value

The perfusion rates retrieved from the dynamic contrast-enhanced MRI scans were 23.8%, 27.0%, 28.0%, 16.2%, and 5.1% for lobes 1–5, respectively. Figure 3 shows the anatomical MRI scan, lobe definitions, and MRI-based activity map after administering five fractions of 250 mg microspheres. Lobes 1–5 received 26.5%, 25.4%, 23.3%, 19.4%, and 5.4% of the total administered activity, respectively, showing a very strong correlation with the perfusion rates (r = 0.932).

**Fig. 4:**
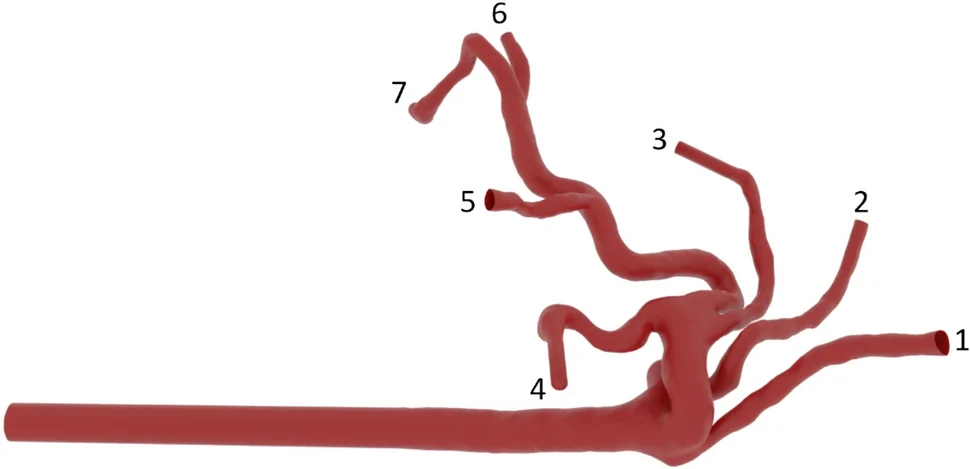
Reconstructed hepatic arterial vasculature, featuring one inlet and seven outlets

Figure 4 represents the reconstructed HA vasculature after truncation. The model featured one inlet and seven outlets, extending to the level where branches supply distinct liver lobes: two supplying lobe 1 (outlet 5 and 7), two supplying lobe 2 (outlet 3 and 6), and one supplying each of lobes 3–5 (outlet 2, 1, and 4 respectively). The vessel inlet was extended by 6.0 cm of straight tubing, and outlets 3 and 4 were extended by 8.0 mm, to promote fully developed flow at the inlets and outlets.

### Part (2) CFD simulations of the ex vivo perfused porcine liver

#### Flow split

**Fig. 5:**
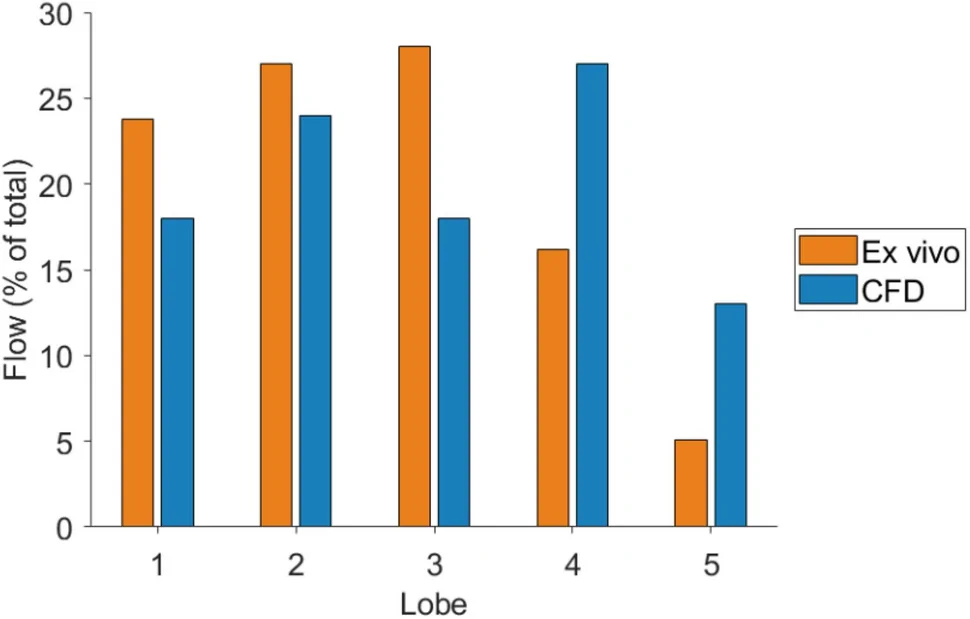
Flow distribution for the ex vivo experiment (based on the dynamic contrast-enhanced MRI scans) and CFD simulations (based on the inlet and outlet boundary conditions)

The flow split for CFD simulations per liver lobe showed fair correlation (r = 0.390) with the ex vivo perfusion rates, with 17.5%, 23.0%, 17.5%, 26.0%, and 15% for lobes 1–5, respectively, see Fig. 5.

#### Catheter configurations

**Fig. 6:**
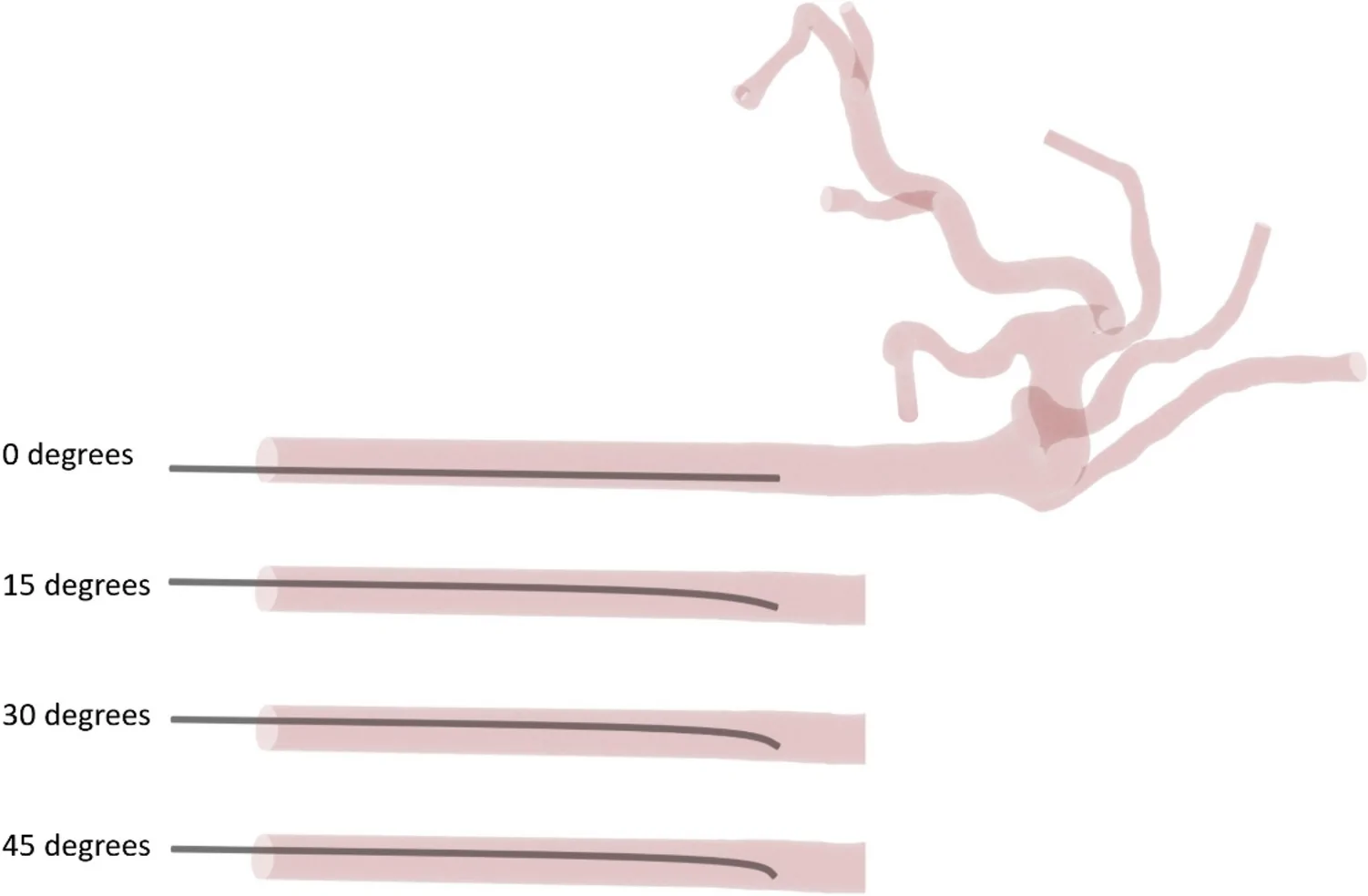
Catheter configurations with different tip angles used in the CFD simulations

After calculating the flow splits, the catheter was positioned in the model, 25 mm from the point of division into five HA branches, replicating approximately the axial catheter position in the ex vivo experiment. Figure 6 illustrates the four different catheter configurations used in the CFD simulations. The catheter tip was maintained at the same radial position across all configurations. Consequently, the catheter shaft was positioned higher in the vessel for configurations with steeper angles.

#### Microsphere injection

**Fig. 7:**
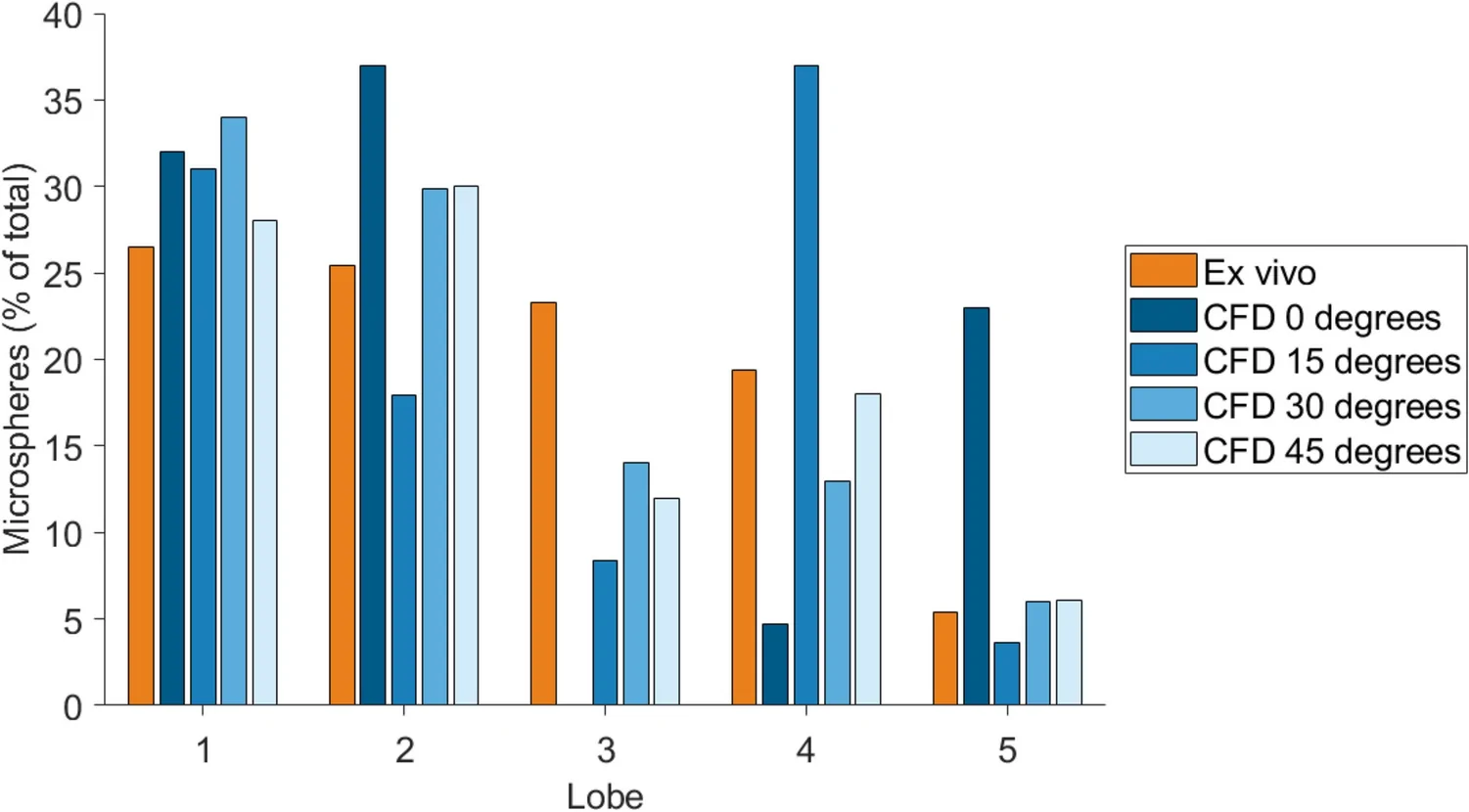
Microsphere distribution per liver lobe in percentages of the total number of microspheres injected, for the ex vivo experiment and the computer simulations under various catheter angles

The distribution of microspheres across the different liver lobes for the ex vivo experiment and the four simulated catheter configurations is presented in Fig. 7. Supplemental File 1 contains videos of the simulations illustrating microsphere trajectories within the vascular geometry. The correlation between the ex vivo microsphere distribution and the CFD simulations was very strong at the 30° angle, though not statistically significant (r = 0.809, *p* = 0.097). Among the four simulated catheter configurations, this angle most closely matched the 25.2° angle observed in the ex vivo experiment. Furthermore, it can be observed that the other catheter configurations led to different microsphere distributions, particularly for the outlets closest to the injection position (lobe 3–5). At 0°, the correlation with the ex vivo microsphere distribution was poor (r = 0.123, *p* = 0.844); at 15°, it was moderate (r = 0.511, *p* = 0.379); and at 30° and 45°, the correlations were very strong (r = 0.809, *p* = 0.097 and r = 0.807, *p* = 0.099, respectively). An increasing catheter tip angle did not consistently result in a stronger correlation across all liver lobes. For instance, in lobe 4, the 15° configuration demonstrated a substantially greater deviation from the ex vivo microsphere distribution compared to the other angles. It is also of interest that in lobe 3 no microspheres reached the outlet in the 0° catheter configuration.

## Discussion

In this study, the impact of catheter angle on the microsphere distribution during TARE was investigated using CFD simulations based on data from an ex vivo machine-perfused porcine liver. Only the controllable parameters obtained from ex vivo experiments were used to parametrize the CFD simulations, to explore the clinical relevance of catheter angle while negating the need for a validation study, which requires incorporation of multiple biological factors.

The CFD simulations highlight the influence of catheter angle on the microsphere distribution. Although the role of an angled-tip microcatheter has been studied before (Aramburu et al. 2017a), the impact of varying catheter angles has not been explored. In our study, we found notable alterations in microsphere distributions, particularly in the outlets closest to the injection position, when the catheter was oriented increasingly angulated toward the HA wall. In contrast, distribution differences across catheter angles were less pronounced in lobes 1 and 2 (with the exception of 15°), which were located further downstream. This may be attributed to the longer transit time toward lobes 1 and 2, allowing microspheres to better align with the blood flow before reaching these outlets (Aramburu et al. 2016a). The deviation observed in the 15° configuration for lobe 4, as well as the absence of microspheres reaching lobe 3 in the 0° configuration, can be attributed to variations in the point of contact between the microspheres and the vessel wall, leading to markedly different trajectories. This effect is highly sensitive to the underlying vascular geometry, underscoring the importance of incorporating patient-specific models to perform CFD simulations.

The similar microsphere distributions observed for catheter angles of 30° and 45° is attributed to the fact that in both configurations, microspheres were directed toward the hepatic arterial wall shortly after leaving the catheter. Interaction with the vessel wall promotes local flow disturbances and enhanced mixing of microspheres with the blood flow, thereby reducing the influence of their initial injection direction. As a result, increasing the catheter angle beyond 30° yielded only a limited additional effect on microsphere distribution.

During the ex vivo experiment, ultrasound imaging confirmed that the catheter tip was oriented toward the HA wall. Even though the ex vivo experiment cannot be directly compared to the CFD simulations, due to differences in flow split (Fig. 5) and injection rate (10 vs 32 ml/min), the ex vivo microsphere distribution and the simulated 30° angle configuration showed very strong correlation (r = 0.809). Among the four simulated catheter configurations, this angle most closely matched the 25.2° angle observed in the ex vivo experiment. This could suggest that injection rate, previously considered a major factor in microsphere distribution (Basciano et al. 2010; Caine et al. 2017; Snoeijink et al. 2025b), may be less influential when microspheres are injected against the HA wall. One possible hypothesis is that the steep injection angle enhances mixing with the blood flow, thereby reducing the impact of injection rate. In other words, effective mixing may already occur at lower injection rates. However, the result was not statistical significant (*p* = 0.097) and could therefore also be due to chance.

The angled position of the catheter against the vessel wall is not incidental. In vessels with significant tortuosity, the resulting tension along the catheter frequently leads to the tip being forced into contact with the vessel wall. Comparable angles have also been observed in clinical practice. (Kao et al. 2011) reported in three clinical cases that catheter angulation against the vessel wall resulted in unpredictable microsphere distributions. Furthermore, previous CFD studies have demonstrated the high influence of the radial catheter position (Basciano et al. 2010; Bomberna et al. 2021; Childress et al. 2012). These studies investigated various release positions relative to the inlet plane’s cross-section to identify optimal positions for directing microspheres to specific outlets.

### Clinical implications

Our ex vivo setup offered the unique advantage of visualizing the catheter angle through ultrasound imaging, by placing the probe directly on the exposed HA. The maximum catheter angle was determined by rotating the ultrasound transducer around the vessel, with the highest angle observed at 25.2°. This level of visualization is not feasible in clinical practice, where catheter placement is typically guided using one-sided 2D planar imaging, leaving the radial orientation of the catheter unknown. This limitation makes it difficult to accurately reproduce catheter positioning between the diagnostic scout dose and therapeutic microsphere dose administration, and this study seems to indicate that small deviations in catheter angle can cause very different microsphere distributions.

A potential solution for improved catheter tip mapping involves acquiring planar images from multiple directions prior to the administration of a scout or therapeutic microsphere dose. This strategy may allow for a more precise determination of the catheter’s radial position. Second, 3D contrast-enhanced CBCT is routinely performed in current clinical practice to visualize the hepatic arterial anatomy during the scout dose. However, the high-contrast bolus injections during these acquisitions can obscure visualization of the catheter tip and its angle. Implementing an additional 3D CBCT acquisition optimized for catheter tip visualization, for example by using lower contrast injection rates, may enhance the assessment of catheter tip positioning. Another solution might be to use alternative catheter designs, such as anti-reflux (van Roekel et al. 2021) or balloon catheters (Mabey et al. 2022), which centers the catheter tip in the arterial lumen during microsphere injection. Nevertheless, further investigation is required to evaluate the efficacy and safety of these catheter types, as complications such as vasospasm and catheter displacement have been reported (van Roekel et al. 2021). Furthermore, (Ortega et al. 2022) developed a multi-side-hole catheter to generate a microsphere distribution that is in better alignment with the flow split to facilitate tumor targeting in their CFD simulations. Ultimately, improved techniques for catheter tip mapping and stabilization may not only enhance the predictive accuracy of the scout dose but also increase the reliability of CFD-based predictive modeling.

### Limitations

A primary limitation of this study is the small sample size (*N* = 1). While the objective was to explore the concept of catheter orientation against the vessel wall, a broader evaluation across diverse vascular geometries is necessary to draw more generalizable conclusions.

Second, the absence of tumors in the ex vivo model and simulations limits clinical translation. Ex vivo, microsphere distribution was found to strongly correlate with the perfusion per liver lobe (r = 0.932). The inclusion of hypervascular tumors would increase flow toward specific segments or liver lobes. It is expected that these areas will attract a greater number of microspheres as well, since in vivo the tumor dose is typically 3 times higher compared to the healthy target dose (Garin et al. 2021; Kühnel et al. 2024). The preferential uptake of microspheres in tumor tissue could potentially reduce the influence of radial catheter positioning on microsphere distribution. However, this hypothesis requires further investigation.

Third, in the ex vivo experiment, the catheter angle was measured only in two 2D planes. Displacements of the ultrasound probe along the vessel’s axial direction (i.e., longitudinal movement) could lead to variations in the measured catheter angle due to local vessel curvature. Nevertheless, a key strength of our study is the recognition that the catheter follows a curved path within the vessel, which is also reflected in the CFD simulations; a unique aspect of our approach. For future research, acquiring the full 3D catheter orientation during the ex vivo experiment is recommended to allow for more detailed analysis.

Several intrinsic limitations of the CFD model should be acknowledged as well. First, the catheter was modeled rigidly, representing an approximation of the actual catheter configuration observed in the ex vivo experiment. Second, the elasticity of the blood vessel walls was not accounted for in the simulations. However, Childress et al. (Childress and Kleinstreuer 2014) investigated this effect and reported only minor differences in microsphere distributions. Additionally, the boundary conditions may not fully capture the physiological conditions, introducing a degree of uncertainty to the model predictions. For example, the perfusion rates per liver lobe, representative for the HA blood flow rates per liver lobe, as derived from the ex vivo experiment, were not fully replicated in the CFD simulations.

Another limitation was the difference in injection rate between the ex vivo experiment and the CFD simulations. A previously established injection rate for sufficient microsphere agitation within the V-vial of the administration device was 24 ml/min (Snoeijink et al. 2025b). In the ex vivo setup, the syringe pumps were connected to the administration device with 3-m tubing to ensure placement outside the 5-gauss safety zone of the MRI scanner. To compensate for pressure loss due to the extended tubing, a slightly increased injection rate was used (32 ml/min). In the CFD simulations, an injection rate of 24 ml/min would have required extremely high spatial and temporal resolutions. The maximum achievable injection rate was 10 ml/min, although previous studies have shown that the injection rate has a major impact on microsphere distribution (Basciano et al. 2010; Caine et al. 2017). This limitation is circumvented by similar limitations in clinical practice. 3D contrast-enhanced CBCT injections are typically administered continuously via an automated contrast media injector at high rates, reaching up to 120 ml/min (May and Charalel 2024), and ^99m^Tc-MAA scout doses are manually injected using a syringe, generally at lower, unquantified rates. In contrast, therapeutic microspheres doses are administered manually through pulsed bolus injections, with individual bolus volumes of approximately 0.1 ml per pulse (Terumo Europe NV, Safety and performance information—Manual—QuiremSpheres™. LC-80072 [05], Issue date: 2023.). This introduces variations in injection rates in clinical practice, of which the impact remains unclear. Future research may benefit from investigating the impact of varying injection rates within CFD simulations of our ex vivo model, as the findings of this study suggest that the influence of injection rate may be diminished due to the microsphere injection against the vessel wall.

Finally, the application of a 0-D Poiseuille flow approximation to model the pressure drop from the inlet to each outlet is a simplification which neglects the effect of the tortuous geometry on the pressure drop, leading to somewhat different flow distributions than those observed ex vivo. Consequently, the CFD model should not be interpreted as an exact digital replica of the ex vivo experiment, and quantitative agreement between CFD-predicted and experimentally observed microsphere distributions should be interpreted with caution. The primary purpose of the CFD simulations was instead to investigate the relative influence of catheter orientation under a consistent set of boundary conditions. Because identical outlet boundary conditions were used for all simulated catheter angles, the observed trends in microsphere distribution remain informative regarding the mechanistic effect of catheter orientation. Similarly, the experimentally measured catheter angle of 25.2° was not explicitly simulated. The CFD simulations were instead designed to evaluate microsphere distributions across a range of catheter orientations (0°, 15°, 30°, and 45°) to assess the sensitivity of microsphere distribution to catheter orientation rather than to reproduce a single experimental configuration. Despite these necessary simplifications, our systematic evaluation of various catheter angles (0°, 15°, 30°, and 45°), gradually directed toward the hepatic arterial wall, is crucial. We contend that by consistently varying this key parameter within our model, the observed trends and the influence of catheter angle on microsphere distribution would remain qualitatively consistent even if a full multiscale model was employed. This approach allows us to identify the fundamental impact of catheter position on distribution patterns, offering valuable and robust insights for clinical practice despite the inherent complexities of the biological system, which can be pursued in future works as progress is made in multiscale models and experimental protocols.

## Conclusions

In conclusion, ex vivo data demonstrated that the catheter may be orientated against the HA wall. CFD simulations investigated the impact of this by varying the catheter angles, and it was found that differences in catheter angle can lead to notable deviations in microsphere distributions. Future research should focus on improving the visualization of the radial catheter position prior to TARE, potentially through optimized use of the planar imaging or 3D CBCT. Additionally, exploring catheter designs that improve positional stability may further support consistent and effective microsphere delivery.

## Supplementary Information

Below is the link to the electronic supplementary material.

Supplementary file1 (MP4 44175 KB)

## Data Availability

The experimental data and the simulation results that support the findings of this study are available from the authors upon request.
